# Zinc Removal from Water via EDTA-Modified Mesoporous SBA-16 and SBA-15

**DOI:** 10.3390/toxics11030205

**Published:** 2023-02-23

**Authors:** Zeinab Ezzeddine, Isabelle Batonneau-Gener, Yannick Pouilloux

**Affiliations:** 1 Institut de Chimie des Milieux et Matériaux de Poitiers (IC2MP)-UMR 7285, Poitiers University, 86073 Poitiers, France; 2 Department of Chemistry, Lebanese University, Beirut P.O. Box 6573, Lebanon

**Keywords:** mesoporous silica, SBA-16, SBA-15, zinc, adsorption, EDTA

## Abstract

The removal of zinc ions from water was investigated using two types of ordered mesoporous silica (SBA-15 and SBA-16). Both materials were functionalized with APTES (3-aminopropyltriethoxy-silane) and EDTA (ethylenediaminetetraacetic acid) through post grafting methods. The modified adsorbents were characterized by scanning electron microscopy (SEM) and transmission electron microscopy (TEM), X-ray diffraction (XRD), nitrogen (N_2_) adsorption–desorption analysis, Fourier transform infrared spectroscopy (FT-IR), and thermogravimetric analysis. The ordered structure of the adsorbents was conserved after modification. SBA-16 was found to be more efficient than SBA-15 owing to its structural characteristics. Different experimental conditions were examined (pH, contact time, and initial zinc concentration). The kinetic adsorption data followed the pseudo-second-order model indicating favorable adsorption conditions. The intra-particle diffusion model plot represented a two-stage adsorption process. The maximum adsorption capacities were calculated by the Langmuir model. The adsorbent can be regenerated and reused several times without a significant decline in adsorption efficiency.

## 1. Introduction

In the past few decades, water contamination has been considered a major problem worldwide. Effluents containing heavy metals such as copper, lead, zinc, and cadmium have been discharged without any treatment into the environment from several industries, such as from steel production, electroplating, and tanneries [1]. The discharged heavy-metal-contaminated wastewater is considered a serious threat to both human health and the ecosystem. Heavy metals are non-biodegradable and may be carcinogenic, thus their presence in water in high amounts could result in critical health issues to living organisms [2,3,4]. Zinc is largely spread in nature and is an essential trace metal for both humans and aquatic organisms [5]. However, if the zinc dosage exceeds a certain quantity, it becomes harmful to organisms [6] as it could interact with biological macromolecules, resulting in a change in their activity and poisoning [7]. Currently, various methods are available for heavy metal removal from water, including membrane filtration, coagulation, precipitation, ion-exchange, and adsorption [8]. The latter is very effective in eliminating heavy metals and is an attractive technique because it does not require complex and expensive installations [9,10]. The adsorption mechanism is defined by the physicochemical properties of the adsorbent and heavy metals and operating conditions (i.e., temperature, adsorbent amount, pH value, adsorption time, and initial concentration of metal ions) [11]. The efficiency of several low-cost adsorbents has been studied, including clay, chitosan, fly ash, zeolites, and activated carbon [12]. Moreover, adsorption is the best method to use when the metal ions’ concentrations are below 100 mg L^−1^ [13]. The adsorption capacity is dependent on the pore size of adsorbents along with the active sites found on their surface [14] and, in recent years, many researchers have focused on developing new adsorbents that meet these criteria. The drawbacks of adsorbents derived from natural materials are their low mechanic resistance to abrasive forces and weak interactions with metal ions [15,16]. One of the advantages of the adsorption process is the ability to modify adsorbents. The carbon surface charges can be enhanced by surface functional groups (such as carboxyl, phenyl, and lactone groups) in order to improve the heavy metal uptake [17]. Surface modification often reduces its surface area and, in turn, increases the content of surface functional groups. Consequently, more metal ions can be adsorbed [18]. The disadvantages of carbon adsorbents are as follows: their high hydrophobicity and the rapid aggregation in aqueous solution owing to large Van der Waals forces, which decreases the adsorption capacity [11]. Chitosan, which is a natural adsorptive polymer, is another adsorbent used, because it has an affinity toward pollutants in wastewaters owing to the presence of amino and hydroxyl groups. However, it suffers from low mechanical strength and poor stability [19]. Mineral adsorbents such as zeolite, silica, and clay are considered good candidates for water purification with low operating costs. Clay has an extraordinary cation exchange capacity (CEC), cation exchange selectivity, surface hydrophilicity, high swelling/expanding capacity, and surface electronegativity [20]. However, their removal efficiency decreases after several sorption cycles. They are also affected by experimental conditions such as pH, irradiation time, adsorbent concentration, wastewater temperature, and the initial dosage of pollutants [21]. In order to overcome these disadvantages, various studies focused on the use of mesoporous silica as adsorbents [22,23], such as MCM-41 [24], SBA-15 [25], and SBA-16 [26]. The ordered structure of such mesoporous silica together with their tailored pore size made them suitable applications for the elimination of pollutants from water [27]. In addition, SBA-16 possesses a three-dimensional structure including cubic Im3m space group with a large pore diameter; dual porosity framework; and large surface area, i.e., a micro porous and mesoporous framework. Such properties make these materials dominant for adsorption [28]. Nevertheless, the surface of these materials has only silanol groups, so it is essential to modify them so that specific binding sites are added. Such chemical modification is achieved by functionalization with different groups, such as -NH_2_ [29]. In addition, mesoporous materials’ modification with chelating was studied as well, because chelating agents increase the metal adsorption capacity. Salicylic acid [30] and cyanex 272 [31] are examples of some of the agents used. The chelating agent ethylene diamine tetraacetic acid (EDTA) is very effective and widely known for heavy metals’ complexion. Moreover, the structure formed between chelating agents and metal ions is stable and they reserve their metal binding properties after chemical regeneration [14].

Herein, SBA-16 and SBA-15 were synthesized, fully characterized, and modified with APTES (3-aminopropyltriethoxy-silane) and then with EDTA. The adsorption capacity for Zn^2+^ removal was then investigated. Moreover, the effect of various parameters on adsorption were studied, including pH, contact time, and zinc concentration. The kinetics of adsorption were also reported using pseudo-first, pseudo-second-order, and intra-diffusion models. The equilibrium isotherms of the experimental data were well fitted by Langmuir isotherm. The regeneration and reuse of the modified adsorbents were performed as well. It is worth mentioning that this manuscript aims to compare the structural impact of both adsorbents on zinc removal, which has not been studied before, other than the effectiveness of these materials as heavy metal adsorbents.

## 2. Materials and Methods

### 2.1. Chemicals

For the template of SBA-16 and SBA-15, Pluronic F127 (EO106PO70EO106) and Pluronic P123 (EO20PO70EO20) triblock copolymers, respectively, were used. Tetraethylorthosilicate (TEOS 98%) was used as the source of silica. For modification, 3-aminopropyltrimethoxysilane (APTES 99%) and ethylenediaminetetraaceticacid disodium salt (EDTA-Na_2_) were utilized. Hydrochloric acid (HCl, 37%), sodium hydroxide (NaOH), toluene, zinc nitrate, and sodium bicarbonate (NaHCO_3_) were also used in this study. All of the reagents were purchased from Sigma Aldrich (St. Louis, MO, USA) and utilized as received without any further purification. Ultrapure water was used throughout.

### 2.2. Adsorbents Synthesis and Modification

**Figure 1:**
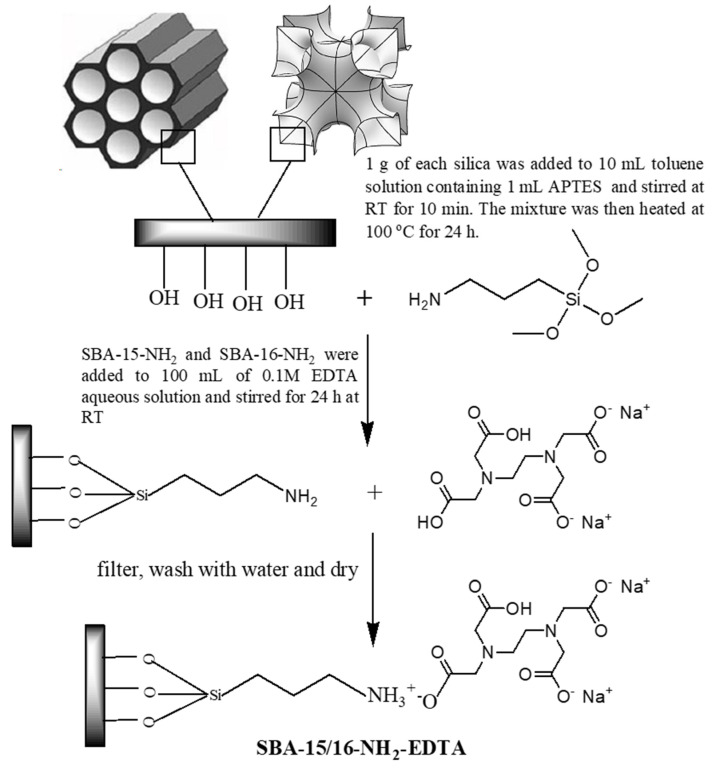
Modification of SBA-16 and SBA-15 with APTES and EDTA.

Mesoporous SBA-16 and SBA-15 were synthesized as described elsewhere in the literature [32,33]. Adsorbents’ modifications were performed through a two-step post synthesis process [34], as illustrated in Figure 1. After APTES and EDTA modification, the adsorbents were denoted as SBA-16-NH_2_ /SBA-15-NH_2_ and SBA-16-EDTA/SBA-15-EDTA, respectively.

### 2.3. Adsorbents Characterization

For determining the textural properties, a Micromeritics TRISTAR sorptiometer (Micromeritics Instrument Corp., Norcross, GA, USA) was used and nitrogen adsorption–desorption isotherms were obtained at −196 °C. The as-synthesized samples were out gassed at 350 °C under vacuum for at least 5 h before measurement and overnight at 150 °C for the modified samples. Low angle X-ray diffraction (XRD) patterns were obtained with an Empyrean X-ray diffractometer (Malvern Panalytical Ltd., Royston, UK) with Cu Kα (λ = 1.54 Å) radiation and a 0.008^o^ min^−1^ rate of scanning between 0.65° and 5° *2*θ. SBA-16 and SBA-15 morphologies were determined by scanning electron microscopy (SEM, JEOL 7001 FEG, Tokyo, Japan) and transmission electron microscopy (TEM, JEOL 2100 UHR at 200 kV, Tokyo, Japan). Thermogravimetric analysis (TG) was conducted using SDT Q600 TA Instrument from 25 to 900 °C in air using SDT Q600 TA Instruments (New Castle, DE, USA). The functional groups were identified in the range of 4000–400 cm^−1^ by Fourier transform infrared spectroscopy (FT-IR–6300 JASCO, Oklahoma City, OK, USA) through mixing the samples with KBr and pressing them into pellets. The amount of immobilized carboxyl groups after EDTA modification was measured by back titration [35].

### 2.4. Batch Adsorption Experiments

The zinc nitrate salt was dissolved in ultrapure water and solutions with different zinc ion concentrations (between 10 and 500 ppm) were prepared. For batch adsorption studies, 20 mg of SBA-16-EDTA or SBA-15-EDTA was added to 20 mL of metal solution of concentration C and the flask was stirred at room temperature (RT) at 300 rpm for 180 min. At the end of each step, the zinc concentration was determined using an atomic adsorption spectrophotometer (AAS, Perkin Elmer AA200, Waltham, MA, USA). The removal efficiency was calculated by Equation (1) [36]:

(1) $$R=\frac{C_{0}\;–C_{t}}{C_{0}}\;\times 100$$

where *C*_0_ and *C_t_* are the heavy metal initial concentration and at concentration at time t, respectively. The adsorption capacity (mg g^−1^) of the adsorbent at equilibrium was calculated by Equation (2) [36]:

(2) $$q_{e}=\frac{\left(C_{0}\;–C_{e}\right)V}{m}\;$$

where *C_e_* is the concentration at equilibrium, (*V*) is the volume in L of metal solution, and m is the mass in g of the adsorbent.

The adsorption isotherms were established by varying the initial metal ion concentrations between 10 mg L^−1^ and 500 mg L^−1^. The solutions were stirred for 180 min at RT and then filtered and the remaining metal ions were measured by AAS in order to calculate *C_e_* and *q_e_*. The pH effect was studied by varying the solution pH between 2 and 8 using 0.1 M HCl or 0.1 M NaOH. Adsorbents’ regeneration was performed with 1 M HCl solution.

## 3. Results and Discussion

### 3.1. Adsorbents’ Characterization

**Figure 2:**
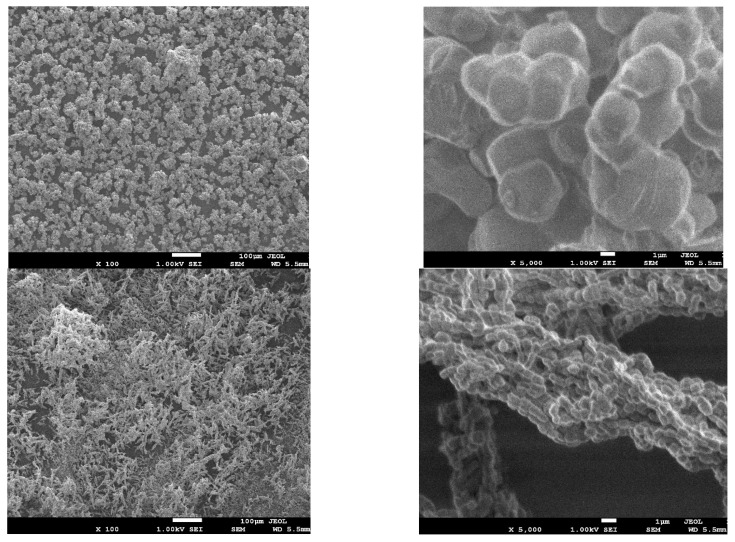
SEM images of of SBA-16 (**above**) and SBA-15 (**below**).

**Figure 3:**
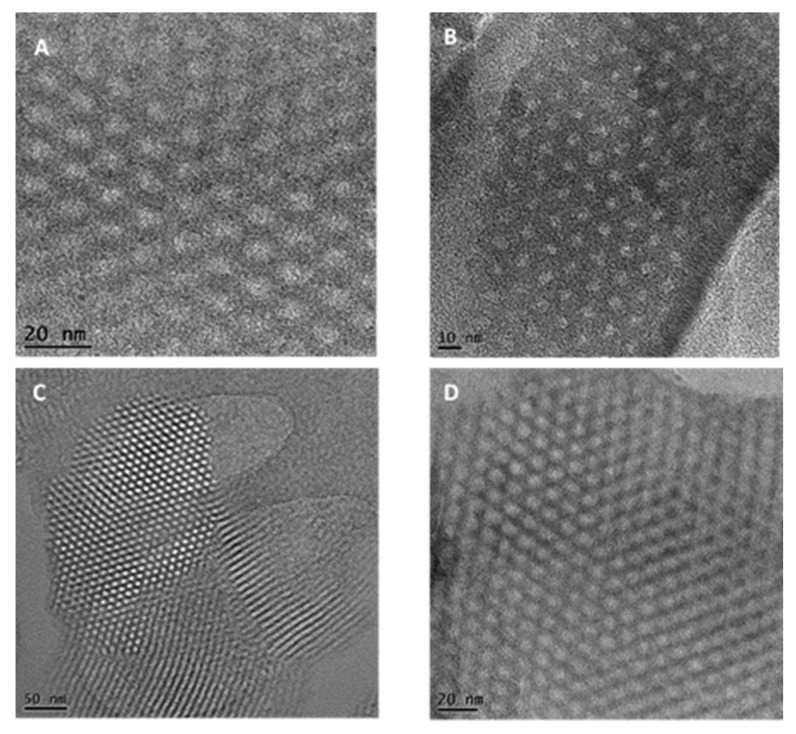
TEM images of SBA-16 (**above**) and SBA-15 (**below**) before (**A**,**C**) and after (**B**,**D**) EDTA modification.

The surface morphologies of the adsorbents were obtained by SEM (Figure 2). SBA-16 appeared as fine cube particles while SBA-15 had rod-shaped particles. The crystallographic structure of both materials was investigated by TEM. SBA-16 images before and after EDTA modification (Figure 3A,C) showed arrays of highly ordered uniform cages demonstrating the 3D cubic structure of SBA-16 that remained unaffected after functionalization. As for SBA-15, Figure 3B,D revealed the highly ordered 2D hexagonal structure (honeycomb structure), which also remained intact after modification.

**Figure 4:**
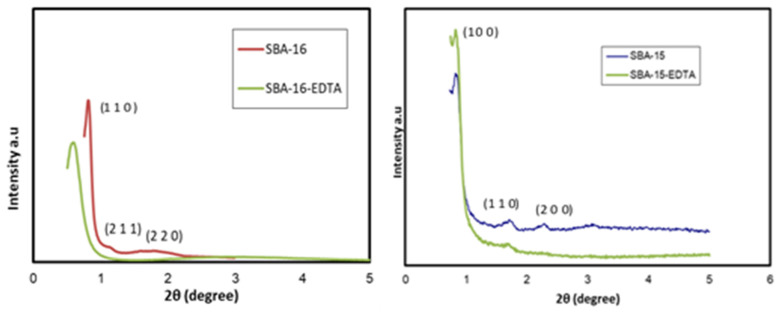
XRD patterns of SBA-16 and SBA-15 before and after modification.

XRD patterns are shown in Figure 4. The peaks in SBA-16 diffractograms corresponding to the (110), (211), and (220) planes, which are indexed at 2θ = 0.8, 1.1, and 1.8, respectively, are characteristics of the cubic body-centered structure (Im3m) [37]. The three diffraction peaks in the SBA-15 pattern, indexed at (100), (110), and (200) planes, are characteristic of the two-dimensional hexagonal symmetry (P6mm) [38]. The three obvious characteristic peaks for SBA-15 are at 2θ = 0.9, 1.7, and 2.2, referring to the (100), (110), and (200) planes, respectively. After EDTA modification, there was a slight pattern shift due to the decrease in pore size, but the symmetrical structure was conserved. For SBA-16-EDTA, the (110) plane shifted to a lower diffraction angle (110) compared with unmodified mesoporous silica SBA-16. Such a shift is due to the decrease in lattice parameters *a* due to pore filling with amino and EDTA functional groups.

**Figure 5:**
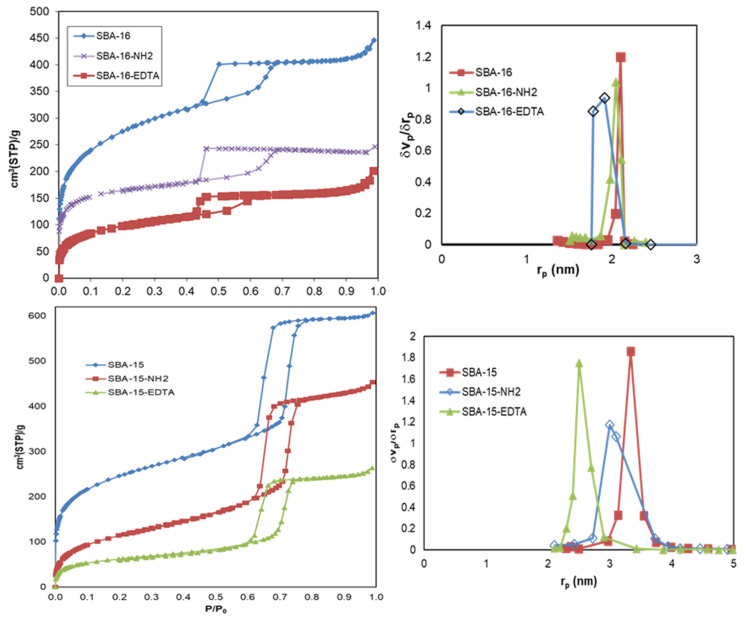
N_2_ adsorption–desorption isotherms and pore size distribution of SBA-16 and SBA-15 before and after modification.

Figure 5 illustrates the nitrogen adsorption–desorption isotherms, and the textural properties and pore size are included in Table 1. According to the IUPAC classification, classical type IV isotherms were made of both materials. SBA-15 exhibited an H1 hysteresis loop, affirming the presence of well-defined and cylindrical mesopores. On the other hand, SBA-16 exhibited an H2 hysteresis loop, which is characteristic of a cage-like mesoporous structure with narrower entrances than the cage itself [39]. For SBA-15, the capillary condensation occurred at a higher relative pressure than for SBA-16, showing that the mesopores of SBA-15 are larger than those of SBA-16. After modification with functional groups, the size of the mesopores along with the surface area and the mesoporous volume decreased for all of the samples. The large decrease in the surface area after modification is mainly the result of the micropores’ blockage by amino propyl groups after modification. As for the shape of the mesopores, no important change was observed after modification and the structure remained intact, which was also proved by XRD and TEM.

**Figure 6:**
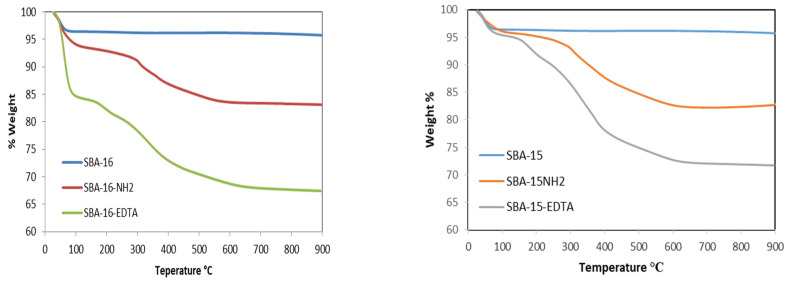
Thermogravimetric curves for SBA-16 and SBA-15 before and after modification.

Thermogravimetric analyses under air were also conducted for the samples before and after modification (Figure 6). The weight loss that occurred below 200 °C is due to the desorption of water. Before modification, the weight losses between 200 and 900 °C are attributed to the silicate networks’ dehydroxylation. For the modified samples, significant weight losses were observed between 200 and 900 °C. Aminopropyl groups were thermally degraded between 100 and 550 °C and the decomposition of EDTA occurred in the same temperature range as well.

**Figure 7:**
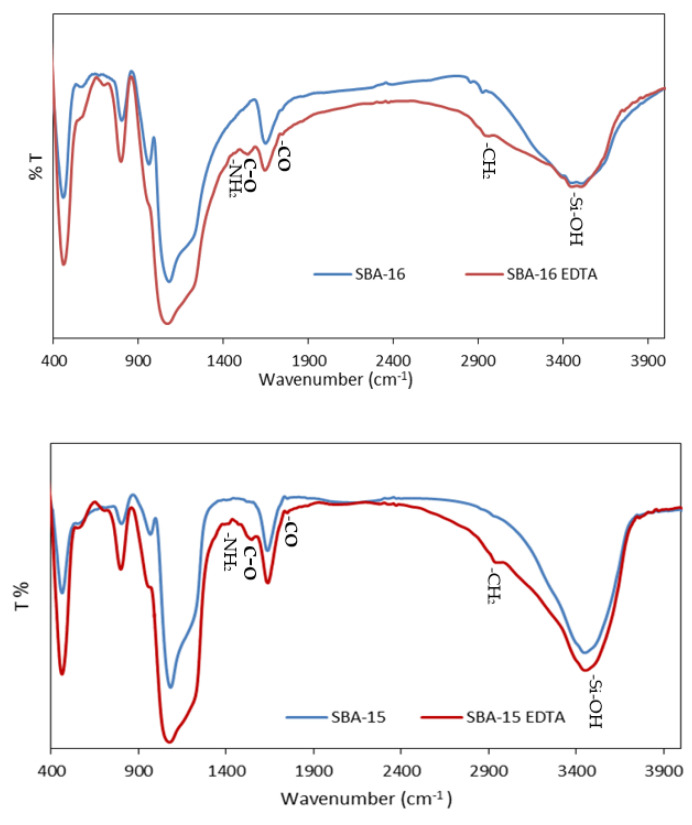
FTIR spectra for SBA-16 and SBA-15 before and after modification.

The infrared spectrum of calcined SBA-15 and SBA-16 (Figure 7) shows typical bands of silanol groups at 3500–3750 cm^−1^ [40]. After modification with aminopropyl groups, the intensity of these bands decreased, while the bands characteristic of aminopropyl groups appeared. These new bands are attributed to the symmetric and asymmetric stretching of CH_2_ groups (*v*_as_ (CH_2_) = 2933 cm^−1^, *v*_s_ (CH_2_) = 2876 cm^−1^), as well as NH_2_ vibration (*v*_as_ = 3372 cm^−1^, *v*_s_ = 3300 cm^−1^) [41]. The band at 1594 cm^−1^ corresponds to NH_2_ bending. The anchoring of EDTA on amino groups resulted in the disappearance of NH_2_ stretching vibration bands at 3372 and 3300 cm^−1^. Moreover, the C-O asymmetrical carboxylate stretching vibration was observed at 1675 cm^−1^. The band at 1744 cm^−1^ was attributed to the stretching vibration of the carboxylic group [42].

**Figure 8:**
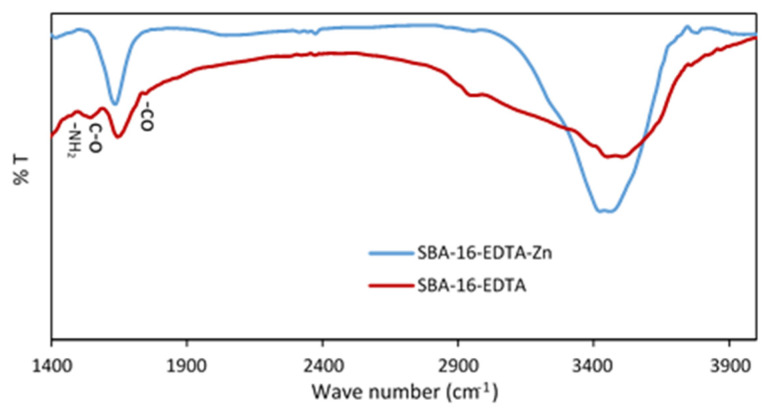
FTIR spectra for SBA-16-EDTA after zinc adsorption.

Figure 8 represents the FTIR spectrum of zinc-loaded SBA-16-EDTA. After zinc adsorption, the band at 1594 cm^−1^ that corresponds to -NH_2_ bending decreased, indicating the adsorption of zinc ions on -NH_2_ groups that did not react with EDTA. Moreover, the bands at 1675 cm^−1^ and 1744 cm^−1^, which correspond to the asymmetrical stretching vibration of the carboxylate C-O and at -CO stretching vibration of carboxylic group, respectively, decreased after zinc adsorption owing to the chelation of zinc ions by EDTA. These results prove the adsorption of zinc ions on SBA-16-EDTA and further prove the heterogeneity of the modified mesoporous silica surface.

### 3.2. Zinc Adsorption Experiments

#### 3.2.1. Effect of Contact Time and pH

**Figure 9:**
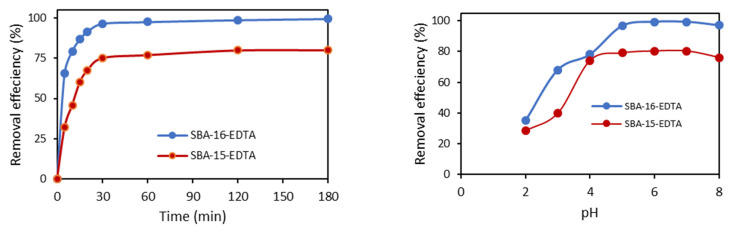
Effect of contact time and pH on Zn^2+^ on EDTA-modified SBA-16 and SBA-15 (at RT and [Zn^2+^]_i_ = 30 ppm).

For both SBA-16 and SBA-15, equilibrium was reached quickly (within the first 30 min) and the amount of Zn^2+^ adsorbed was much higher for SBA-16 (Figure 9). The obtained results may be due to the difference in structure between SBA-15 and SBA-16. The latter cage-like structure favors the diffusion of zinc ions.

The pH of the solution directly affects zinc ion adsorption because it controls its speciation as well as the adsorbent surface charge. The removal of Zn^2+^ ions on both adsorbents increased as pH increased from 2 to 7 (Figure 9). As the pH increased from 2 to 4, the adsorption efficiency increased from 35% to 78%. At pH 5, the adsorption further increased to 96.6% and reached 99% at pH 6 and 7. Above pH 7, the adsorption capacity slightly decreased (97%). For pH values higher than 3, EDTA molecules have a carboxylate form, thus increasing zinc complexation. Above pH 7, zinc starts to precipitate and form complexes with OH^−^ (Zn(OH)_2_).

#### 3.2.2. Adsorption Kinetics

The two kinetic models, pseudo first-order and pseudo-second order, used to calculate the kinetic parameters are expressed in Equations (3) and (4), respectively [43]:

ln (*q*_e_ − *q*_t_) = ln *q*_e_ − *k*_1_t (3)

(4) $$\frac{t}{q_{t}}=\frac{1}{k_{2}q_{e}^{2}}+\frac{t}{q_{e}}$$

where *q_t_* and *q_e_* are the quantity of metal ions adsorbed (mg g^−1^) at time t (min) and at equilibrium, respectively. *k*_1_ (min^−1^) and *k*_2_ (g mg^−1^ min^−1^) are the pseudo-first- and pseudo-second-order rate constants.

**Figure 10:**
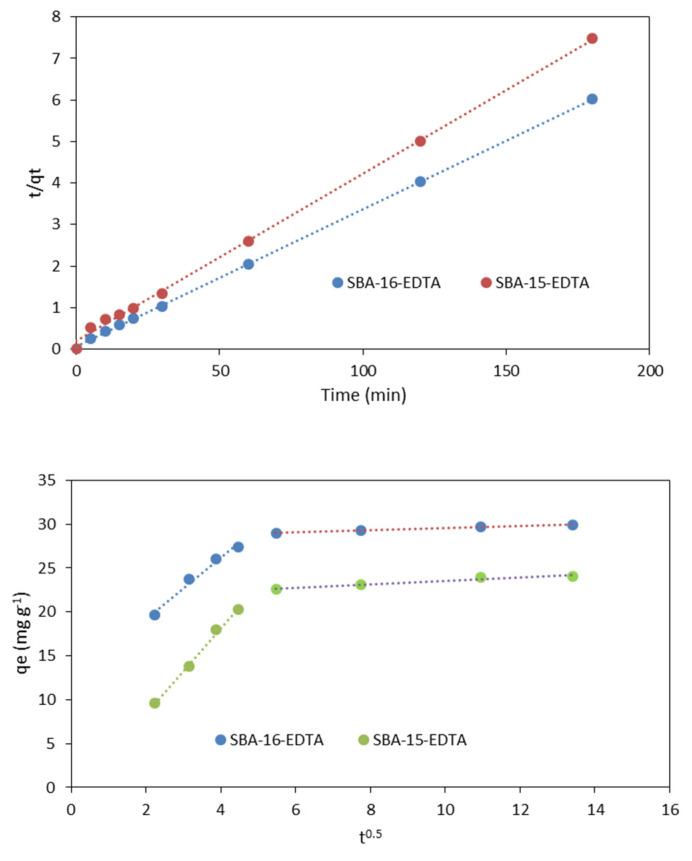
Pseudo-second-order model kinetic model (**above**) and intra-particle diffusion model (**below**) for Zn^2+^ adsorption (pH = 6 at RT).

The theoretical qe values obtained from the pseudo-second-order kinetic model were very close to the experimental ones (Table 2). The obtained results indicated that zinc ion adsorption on both adsorbents followed the pseudo-second-order kinetic model (Figure 10). This model is based on sorption equilibrium capacity and presumes that the sorption capacity is proportional to the number of active sites occupied on the sorbent [44]. This suggests that the adsorption rate mainly depends on the active adsorption site content on the adsorbent surface, and the rate-limiting step is chemisorption involving valence forces through sharing or exchange of electrons between specific sites on adsorbent and metal ions [45]. The chemical interaction between EDTA and metal ions is correlated and in accordance with the kinetic results obtained.

To determine if the intra-particle diffusion is a rate-limiting step in the zinc adsorption on both adsorbents (SBA-15 and SBA-16), the intra-particle diffusion model proposed by Weber and Morris [46] was used to analyze the kinetic results. This model is expressed as follows:

*q*_t_ = *K*_id_t^1/2^ + C (5)

where *k*_id_ is the rate constant of intra-particle diffusion (mg g^−1^ min^−1/2^) and C is the intercept (mg g^−1^). High C values propose that external diffusion has a greater role than the rate-limiting step, because the C value is related to the boundary layer thickness [47]. A plot of the zinc amount adsorbed (*q*_t_) versus t^0.5^ should be linear, and if the line passes through the origin, then intra-particle diffusion is the only rate-controlling step [48]. The obtained results are illustrated in Figure 9 and the parameters are displayed in Table 3. The plots present two linear parts indicating that two steps have occurred. The first sharp part corresponds to the external surface adsorption, while the second part represents the gradual adsorption step, such that the intra-particle diffusion is rate-limiting [49]. As the plot *q_t_* versus t^0.5^ was not a straight line passing through the origin, the process of adsorption is not controlled only by the intra-particle diffusion where film diffusion might have an effect on the kinetics.

#### 3.2.3. Adsorption Isotherms

The adsorption behavior for both adsorbents was analyzed by Langmuir and Freundlich isotherm models in order to model the amount of solute adsorbed per unit of adsorbent, *q_e_*, as a function of equilibrium concentration in the bulk solution, *C_e_*, at a constant temperature. The Langmuir and Freundlich, models, are expressed in Equations (6) and (7), respectively:

(6) $$\frac{C_{e}}{q_{e}}=\frac{1}{K_{L}q_{max}}+\frac{C_{e}}{q_{max}}$$

(7) $$\log q_{e}=\log K_{f}+\frac{1}{n}logC_{e}$$

where *C_e_* and qmax denote the metal concentration (mg L^−1^) at the equilibrium state and the adsorption capacity (mg g^−1^), respectively. The value of *n* is the inverse of the heterogeneity factor of the adsorption process. Meanwhile, *K_L_* and *K_f_* are the Langmuir (L mg^−1^) and Freundlich (mg g^−1^) constants related to the mean free energy of adsorption, respectively [50,51].

**Figure 11:**
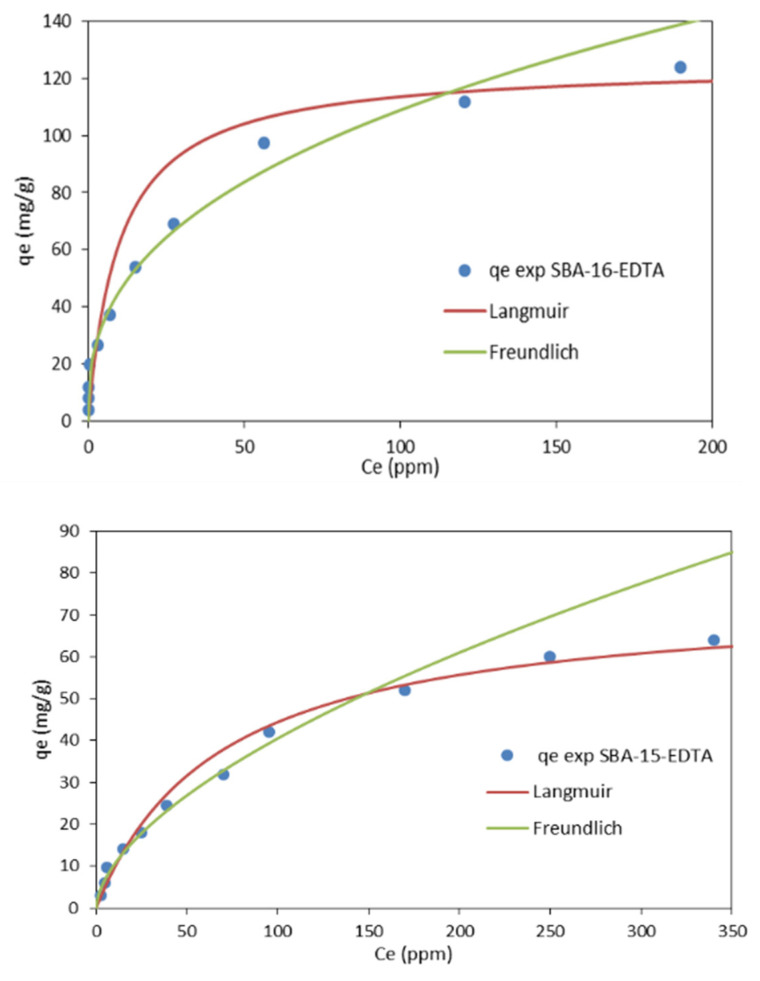
Langmuir and Freundlich adsorption isotherms for Zn^2+^ adsorption on EDTA-modified SBA-16 and SBA-15.

The adsorption isotherms of the experimental data are shown in Figure 11 and the parameters of these two models are shown in Table 4. From the linear regression correlation coefficient R^2^, it can be deduced that the equilibrium data could be well described by the Freundlish isotherm, so the adsorption is reversible in a heterogeneous system that is not limited to the formation of monolayers [52]. The values of *n* were all between 1 and 10, indicating that the adsorption performance of zinc ions on both adsorbents was favorable under the studied conditions [53], so both adsorbents can be considered efficient for zinc metal ion removal, with the preference for SBA-16. Moreover, the Freundlich expression is an exponential equation and, therefore, assumes that, as the metal ions’ concentration increases, their concentration on the adsorbent surface also increases, indicating a non-ideal adsorption, not limited to monolayer formation.

Contrary to the Langmuir isotherm model, which is commonly used for monolayer adsorption, where most of the adsorption sites have equal affinities toward the asorbate, the Freundlich isotherm model is used to describe a heterogeneous chemisorption process in which the surface is not energetically uniform [54]. Isotherms of this form have been observed for a wide range of heterogeneous surfaces, including activated carbon, silica, clays, metals, and polymers. In the case of SBA-16-EDTA and SBA-15-EDTA, the obtained results showed that the data fitted Freundlich, as previously mentioned, and this is mainly because of the heterogeneous surface of both adsorbents, taking into consideration the presence of some unfunctionalized silanol groups, amino groups along the EDTA fixed on the majority of sites. Such heterogeneity will directly govern the adsorption process on both adsorbents and, as a result, the obtained isotherm as well. It can be concluded that Freundlich fits well over the entire concentration range; however, there is an obvious deviation at higher concentrations. So, in general, the Freundlich isotherm will be a more accurate approximation at lower concentrations [55]. Moreover, the Freundlich expression is an exponential equation and, therefore, assumes that, as the metal ions’ concentration increases, their concentration on the adsorbent surface also increases, indicating a non-ideal adsorption, not limited to monolayer formation [56]. Concerning the chemisorption process, the presence of functional groups can be used as evidence in proposing the adsorption mechanism. The Zn-chemisorption mechanism onto EDTA-modified SBA-16 and SBA-15 can be proposed as follows: Zn^2+^ ions bind to carboxylate groups of EDTA via ionic forces with carboxylic oxygen atoms. These oxygen atoms exhibit a negative charge in their structure as a result of the dissociation of carboxylic groups. The negatively charged oxygen atom in carboxylate anions will coordinate with zinc cations, resulting in the formation of metal–carboxylate complexes (COO–Zn) on the adsorbents’ surface [57,58].

## 4. Adsorbents’ Regeneration

**Figure 12:**
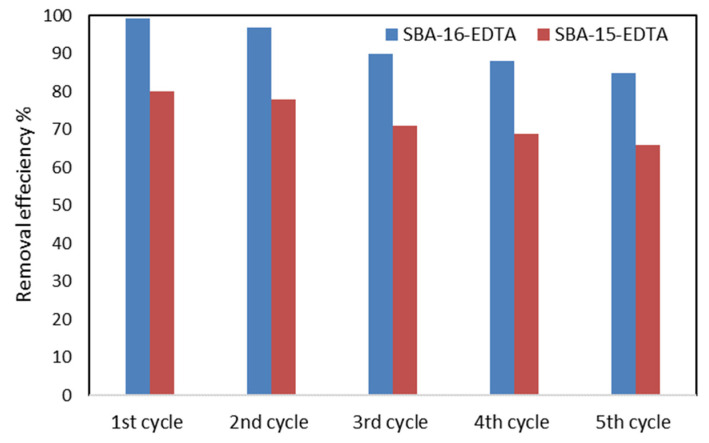
Regeneration and reuse of SBA-16-EDTA and SBA-15-EDTA.

Batch desorption experiments and the desorption efficiencies were compared for both adsorbents. In two beakers, each containing 40 mL of 1 M HCl and 20 mg of SBA-15-EDTA or SBA-16-EDTA at RT, the mixtures were stirred at 300 rpm for 2 h. Then, the mixtures were filtered and dried in order to be used again. Five regeneration cycles were performed. Figure 12 shows that, after the fifth regeneration cycle, both adsorbents conserved about 85% of their removal efficiency.

## 5. Conclusions

In this study, two adsorbents were prepared by EDTA immobilization on SBA-15 and SBA-16 mesoporous silica. The ordered mesostructures of the obtained hybrid organic/inorganic materials were well preserved after modification. The modified adsorbents were characterized by scanning electron microscopy (SEM) and transmission electron microscopy (TEM), X-ray diffraction (XRD), nitrogen (N_2_) adsorption–desorption analysis, Fourier transform infrared spectroscopy (FT-IR), and thermogravimetric analysis. The effects of contact time and pH on zinc adsorption on both materials were studied. The adsorption process was fast, indicating the high affinity of adsorbents to chelating zinc ions. The modified SBA-16 showed higher efficiency for eliminating Zn^2+^ compared with SBA-15 owing to its favorable structure characteristics (pore structure). The kinetic data well fitted the pseudo-second-order model, where the rate adsorption process depends on the exchange kinetics between the ligand and zinc ions. The effect of intra-particle diffusion was also investigated. Equilibrium data were also fitted by Freundlich isotherm. Both adsorbents can be regenerated using HCl solution and reused for up to five cycles.

## Figures and Tables

**Table 1:** Textural properties of SBA-16 and SBA-15 before and after modification.

Sample	S_BET_^a^ (m^2^ g^−1^)	Pore Size ^b^ (nm)	Mesopore Volume ^c^ (cm^3^ g^−1^)	Micropore Volume (cm^3^ g^−1^)
**SBA-16**	954.8	4.43	0.485	0.122
**SBA-16-NH_2_**	567.8	4.14	0.223	0
**SBA-16-EDTA**	330.5	3.98	0.189	0
**SBA-15**	860.3	6.81	0.782	0.104
**SBA-15-NH_2_**	422.2	6.01	0.587	0
**SBA-15-EDTA**	229.03	4.76	0.336	0

^a^ S_BET_ is the BET surface area evaluated in the range of relative pressures p/p_o_ of 0.05–0.2. ^b^ Pore diameter calculated using the BJH method. ^c^ Total pore volumes were calculated by converting the amount adsorbed at p/p_o_ ~0.99.

**Table 2:** Comparison of the first- and the second-order kinetic models for Zn^2+^ adsorption.

	*q_e_* ^exp^ (mg g^−1^)	First-Order Kinetic Model		Second-Order Kinetic Model			
		*k*_1_ (min^−1^)	*q_e_*^cal^ (mg g^−1^)	R^2^	*q_e_*^cal^ (mg g^−1^)	*k*_2_ (g mg min^−1^)	R^2^
SBA-16-EDTA	29.9	0.031	5.7	0.826	30.3	0.014	0.999
SBA-15-EDTA	24	0.102	15.83	0.992	24.7	0.008	0.998

**Table 3:** Parameters of the intra-particle diffusion model.

	k_id1_ (mg g^−1^min^−1/2^)	C_1_ (mg g^−1^)	R^2^	k_id2_ (mg g^−1^min^−1/2^)	C_2_ (mg g^−1^)	R^2^
SBA-16-EDTA	3.5	12.2	0.981	0.114	28.4	0.991
SBA-15-EDTA	4.8	-	0.994	0.196	21.5	0.925

**Table 4:** Langmuir and Freundlich models for Zn^2+^ adsorption on SBA-16 and SBA-15.

		Langmuir Model			Freundlich Model		
	*q^exp^_max_* (mg g^−1^)	K_L_ (L mg^−1^)	R^2^	*n*	K_f_ (mg g^−1^)		R^2^
SBA-16	184.1	0.1	0.964	3.32	25.12		0.974
SBA-15	107	0.03	0.682	1.28	4		0.985

## Data Availability

Authors ensure that this manuscript is ethically sound and meet industry-recognized standards that are reflected in MDPI policies.
